# Improving lifestyles sustainability through community gardening: results and lessons learnt from the JArDinS quasi-experimental study

**DOI:** 10.1186/s12889-020-09836-6

**Published:** 2020-11-26

**Authors:** Marion Tharrey, Ashby Sachs, Marlène Perignon, Chantal Simon, Caroline Mejean, Jill Litt, Nicole Darmon

**Affiliations:** 1 MOISA, Univ Montpellier, CIRAD, CIHEAM-IAMM, INRAE, Institut Agro, Montpellier, France; 2 University of Colorado Boulder, Boulder, CO USA; 3 CarMen Laboratory, INSERM 1060, INRA 1397, University of Lyon, F-69600 Oullins, France; 4 ISGlobal, Barcelona, Spain

**Keywords:** Quasi-experiment, Health promotion, Food supply, Nutrition, Physical activity, Accelerometer, Well-being, Connection to nature, Urban garden, Mental health

## Abstract

**Background:**

Despite an increasing number of studies highlighting the health benefits of community gardening, the literature is limited by cross-sectional designs. The “JArDinS” quasi-experimental study aimed to assess the impact of community garden participation on the adoption of more sustainable lifestyles among French adults.

**Methods:**

Individuals entering a community garden in Montpellier (France) in 2018 (*n* = 66) were compared with pairwise matched individuals with no experience in community gardening (*n* = 66). Nutritional quality, environmental impact and cost of monthly household food supplies, level of physical activity measured by accelerometers, as well as mental and social well-being, sensitivity to food waste, and connection with nature were evaluated at baseline (t0) and 12 months later (t1) to explore sustainability of lifestyles in social/health, environmental and economic dimensions. Linear mixed models were used to determine the independent effect of community gardening on investigated lifestyles components. In-depth interviews were conducted at t1 with 15 gardeners to better understand changes that may have occurred in gardeners’ lives during the first year of gardening.

**Results:**

At t0, gardeners had lower education level, lower BMI and their household reported lower percentage of meals consumed outside of the home compared to non-gardeners (*p* <  0.05). Participating in the community garden had no significant impact, in spite of sufficient statistical power, on fruit and vegetables supplies (main outcome), nor on physical activity parameters, nor on others of the social/health, environmental and economic lifestyles components investigated. Qualitative interviews suggested the existence of pre-established health and environmental consciousness in some gardeners and revealed several barriers to the participation such as lack of time, lack of gardening knowledge, physical difficulty of gardening, health problems and conflicts with other gardeners.

**Conclusions:**

The health benefits of community gardening previously reported by cross-sectional studies might be confounded by selection bias. The JArDinS study highlights the need to identify solutions to overcome barriers related to community garden participation when designing relevant public health interventions for the promotion of sustainable lifestyles.

**Trial registration:**

The study was registered at clinicaltrials.gov as NCT03694782. Date of registration: 3rd October 2018, retrospectively registered.

**Supplementary Information:**

The online version contains supplementary material available at 10.1186/s12889-020-09836-6.

## Background

In recent years an increasing body of literature has suggested that gardening could address various public health concerns through its positive effects on nutrition, physical activity, social cohesion, quality of life, stress and depression [1]. Beyond evidence drawn from gardening programs within institutions such as schools or health care settings [2–5], less is known about the potential health effects of urban collective gardening on  free-living adults. Collective gardens, defined as cultivated spaces managed collaboratively by groups of gardeners located at a distant place from gardeners’ homes, generally take the form of community or allotment gardens [6]. While allotments are large plots of lands usually located in peri-urban areas, rented to a person or a family for cultivation purposes [7, 8], community gardens are smaller plots of land usually integrated in the fabric of urban neighbourhoods, grown collectively [9]. The cost of the annual subscription varies from a hundred euros to a few euros, depending on the type of garden (allotment or community) and the type of land (private or public). Primarily intended to favour social links and intergenerational exchanges among the inhabitants of a neighbourhood, community gardens are a popular way to engage with green spaces, as they provide urban dwellers an avenue to access nature and safe and healthy food. Pathways through which community gardens could amplify individual health include intrapersonal, inter-personal and environmental processes, such as self-efficacy, attitudes, motivation, social support, neighbourhood attachment or aesthetic [10]. Yet, while several studies have shown a positive association between community gardening and fruit and vegetable consumption [11–15], inconclusive results were found for BMI [15–18], physical activity [12, 15], social health [12, 14, 15, 19, 20] and mental well-being [12, 15]. The lack of conclusive evidence regarding differences in health related behaviors and health status between gardeners and non-gardeners is partly due to methodological limitations of existing studies including the use of cross-sectional designs, convenience samples, small sample sizes, and self-reported measurements of health outcomes [10, 21]. Alaimo et al. particularly encouraged the next generation of gardens’ research to take advantage of so called “natural experiments” (i.e. interventional researches in which the experimental conditions are self-determined without being manipulated by researchers) to evaluate the health impacts of community gardens [10]. While several randomized controlled trials on home or community gardening are under way in the United States [22–25], natural experiments offer another opportunity of longitudinally assessing the changes induced by community gardening [26].

Beyond health-related outcomes, shaping behavioral patterns from a sustainability perspective could lead to more sustainable lifestyles [27]. Community gardening, for example, could raise gardeners’ environmental awareness and encourage the adoption of more sustainable dietary practices by fostering collective thinking about biodiversity and eco-friendly practices [28]. Additionally, by providing access to fresh food harvested from the garden, community gardens could favor food affordability by reducing food expenses [29] or changing purchasing behaviors [30]. Community-based interventions targeting gardening thus appear as a relevant tool to positively influence the three fundamental pillars of sustainability - namely social (including health outcomes), environment and economy [31] – and therefore, promote more sustainable lifestyles. The term “lifestyles” is commonly used in public health to define a cluster of habits that include an individual’s behaviors, inclinations, preferences and values that affect health status [32]. The present study was aimed at assessing the impact of urban community garden participation on the adoption of more sustainable lifestyles on  free-living adults in a European context. We used both qualitative and quantitative approaches to better explore changes that may have occurred in gardeners’ lives during the first year of gardening, and the potential benefits to their physical, social and mental health.

## Methods

### Study setting, population and design

JArDinS is a quasi-experimental study conducted between 2018 and 2019. The design and protocol of the study have already been described in a previous paper [33]. A convenience sample of new community gardeners (herein referred to as “gardeners”) was established in 2018 (experiment group). All known community gardens (*n* = 34) in Montpellier (France) were contacted and informed about the study. Garden plots were grown either collectively or individually (≤ 20 m^2^ for individual plots). Gardens with collective plots were accessible to anyone who joined the garden organization and paid the annual dues. For gardens with individual plots, gardeners usually registered on a waiting list. Throughout the gardening season (from March to November), each new individual entering one of the contacted gardens was invited to participate in the JArDinS study. Inclusion criteria were as follows: 1) starting gardening in a community garden, 2) being willing to be involved in the study for 1 year, 3) be at least 18 years old, 4) being able to read French, and 5) residing in the city of Montpellier. Exclusion criteria were: 1) past experience of at least one household member in community gardening, 2) self-reported extensive experience in gardening, 3) never shopping for household food supply, and 4) having a chronic disease. In parallel, a matched-control group of non-gardeners was formed by selecting volunteers participating in a population-based survey on food supply behaviours in Montpellier (“Mont’Panier” survey). To be selected, participants from the control group must neither garden, nor have had prior experience in a community garden or have planned to join one. Matching criteria were: age (< 30; 30–50; > 50 years old), gender, household income (< 1110; 1110–1999; 2000–2699; ≥ 2700 € per month and per consumption unit) and household composition (single adult with no child; single adult with at least 1 child; > 1 adult with no child; > 1 adult with at least 1 child). Data were collected at baseline (t0) and 12 months later (t1). Household fruit and vegetables supply was the main outcome of the study. A total sample size of 160 participants (80 gardeners and 80 non gardeners) was previously evaluated to detect an increase of one portion of fruit and vegetables per day and per person in the gardeners group with 80% power at the 0.05 level of significance and a planned attrition rate of 30% [33].

The study was conducted in accordance with the Declaration of Helsinki, and the protocol was approved by the Ethics Committee of the French Institute for Health and Medical Research and the Commission Nationale Informatique et Libertés (IRB00003888); verbal informed consent was obtained following recommendation of the Research Ethics Committee. The JArDinS study was registered at clinicaltrials.gov as NCT03694782. Participants received a 15 € voucher at t0 and at t1 for returning all data collection materials duly completed.

### Quantitative evaluation

#### Data collection and lifestyles sustainability assessment

Details about collection and assessment of all outcome variables of the JArDinS study have been summarized in a previous methodological paper [33]. To investigate the social/health, environmental and economic dimensions of sustainability, we instructed participants to 1) complete a 1-month food supply diary and collect food receipts, 2) wear a hip-worn triaxial accelerometer (wGT3X-BT or wActiSleep-BT, Actigraph, Pensacola, FL, USA) for 9 consecutive days and 3) fill in an online questionnaire including self-reported body height and weight, and validated questionnaires on mental well-being, social health, sensitivity to food waste and connection with nature.

The social/health dimension was approached by measuring: 1) the healthiness of household’s food supply based on *i)* fruit and vegetables household supply (including fruit and vegetables from the garden) in gram consumed per day and per person living in the household and being concerned by the food supplies (see Additional file 1); *ii)* two indicators of nutritional quality: the mean adequacy ratio (MAR) which assesses the percentage of adequacy in relation to 20 essential nutrients [34], and the mean excess ratio (MER), which assesses the excess intake of sodium, free sugars and saturated fatty acids [35]; *iii)* the Healthy Purchase Index (HPI) estimating the healthiness of household food purchases, based on food expenditure only [36]; 2) participant’s physical activity energy expenditure (PAEE) and time spent during daytime in inactivity (< 1.5 METs), light intensity activities (between 1.5 and 3 METs) and moderate-to-very vigorous intensity activities (> 3 METs) using a previously validated model that combines an automatic activity-recognition algorithm with an activity-specific count-based model [37]; 3) self-reported BMI; 4) mental well-being (WEMWBS) [38]; and 5) level of social isolation (UCLA Loneliness Scale, V3) [39]. Additional file 1 further describes specific details regarding the data analyses of food supply diary and accelerometer.

The environmental lifestyles dimension was assessed through key indicators of food practice sustainability, namely 1) the greenhouse gas emissions (GHGE, in g CO_2_eq), atmospheric acidification (in g SO_2_eq), marine eutrophication (in g Neq) and animal to plant protein ratio of household food supply; 2) participants’ sensitivity to food waste (Sensitivity to food waste scale) [40]; 3) and their connection with nature (Nature Relatedness Scale) [41]. Because of the skewed distribution of the sensitivity to food waste score participants were classified in two categories: “high sensitivity to food waste” (score ≥ median at t0) or “low sensitivity food waste” (score < median at t0).

The economic dimension was approached by household food expenditure and expenditure share by food groups. For food coming from the garden, or from gifts or food aid, a theoretical expenditure was attributed based on the mean observed food price for that product in the rest of the sample.

#### Additional information

The online questionnaire (see Additional file 2) further provided information on socioeconomic characteristic of participants, percentage of meals consumed outside of the home in total household meals, participant’s exclusion of selected foods and perceived competence in gardening (“beginner”, “intermediate”, “advanced”), as well as a social desirability scale (the Balanced Inventory of Desirable Responding Short Form) [42]. The questionnaire for gardeners also contained a specific section on the community garden to collect information on the characteristics of the garden, garden-to-home distance and transportation used to go to the garden at t0; and periods of inactivity in the garden during the past year (ranging from none to > 9 months), as well as frequency of gardening during activity periods (“≥ 1 time/week”,“1–3 times/month”, “< 1 time/month”) at t1.

To compare our results to gardeners’ perception of changes that took place in their lives, we sent them an online post-survey questionnaire, in which gardeners were asked if they had perceived a change in their fruit and vegetable consumption, physical activity, life satisfaction and social relation during the past year (five response categories: “strong increase”, “slight increase”, “no change”, “slight decrease” or “strong decrease”) and if that change was due to the community garden (Additional file 2).

### Statistical analyses

Sociodemographic characteristics and key outcome variables of participants at baseline were compared between the two groups (gardeners vs non-gardeners) using paired t-test for continuous variables and McNemar test for categorical variables. The changes in the lifestyles sustainability components between the two groups across time (pre- to post-test) was investigated only for remaining gardener-control pairs at t1. We used linear mixed-effect models (SAS PROC MIXED) for continuous variables and logistic mixed-effect models (SAS PROC GLIMMIX) for dichotomous variables. Group, time and the time*group interaction were treated as a fixed effect. We modelled within-person variation by using a compound symmetry covariance matrix. Significant time*group interaction indicated a difference in outcome over time between the two groups. Different adjustments were used depending on the outcome variable. Models on physical activity were adjusted for baseline education level and BMI (Model A). Models on food supply and models on self-report data were further adjusted for percentage of meals consumed outside of the home (Model B), and social desirability scale (Model C), respectively. Model on BMI was adjusted for baseline education level, percentage of meals consumed outside of the home and social desirability scale (Model D). Indicators with skewed distribution were log-transformed to improve heteroscedasticity and improve normality of the residuals. Post-hoc analyses were performed only on active gardeners (who visited the garden at least once a month throughout the year) and on those who did not drop out the garden during the year.

We performed all analyses with the SAS statistical software package Ver. 9.4 for Windows (SAS Institute, Cary, NC, USA), with statistical significance at *p* <  0.05.

### Qualitative evaluation

The qualitative evaluation was based on interviews with 15 community gardeners in the study conducted over 7 weeks in May and June 2019. We selected a sample size of 15 as it allowed us to select individuals from geographically and socioeconomically varied community gardens. The interview sample included 11 females and 4 males. All interviews were conducted and recorded in French and later professionally transcribed into English for analysis using an online translation and transcription service. We held interviews in the participants’ homes or community gardens, whichever was more convenient. Interviews ranged from 11 to 50 min. Using a semi-structured interview guide, we led interviews with 5 to 7 open-ended questions directed at the new gardener experience to better understand changes that may have occurred in gardeners’ lives during the first year of community garden participation. We checked each transcript carefully for accuracy against the recordings after receiving it from the transcription service. Assisted by ATLAS.ti qualitative software, we followed grounded theory methodology by analysing the data inductively without a predetermined codebook [43]. We selected key concepts by extracting repetitive topics in the data for closer analysis, sorting codes into larger code groups, and visually networking these groups to distil salient themes [44].

## Results

### Participant eligibility and sample size

**Fig. 1:**
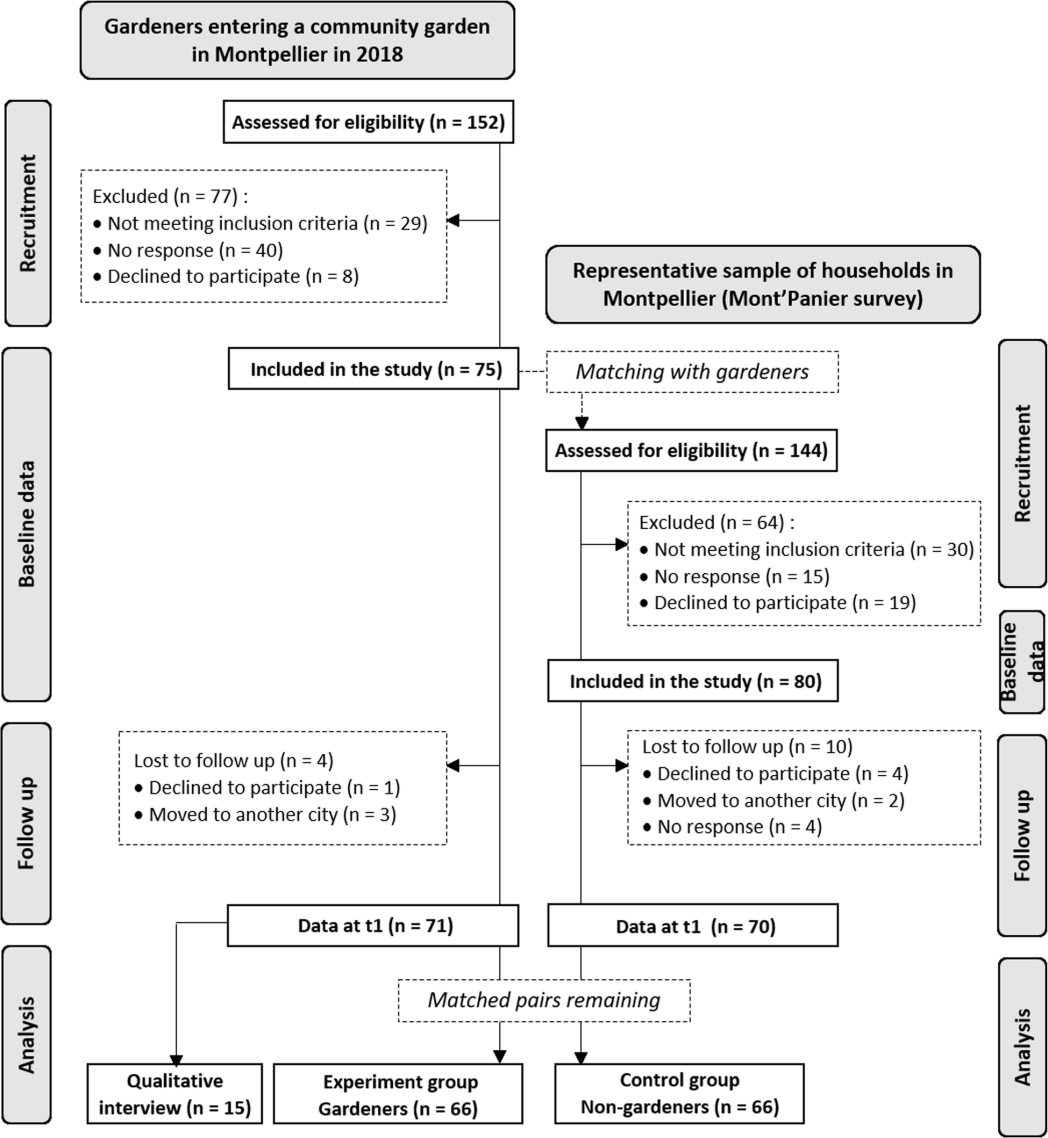
Flow diagram of the JArDinS study

In total 296 potential participants were approached between March and November 2018 (152 gardeners starting gardening in a community garden and 144 matched non-gardeners). Fifty-nine were excluded, as they did not meet the inclusion criteria and 85 declined to participate or did not answer resulting in a final sample of 155 participants at baseline (response rate: 61.0% for gardeners and 70.2% for controls) (Fig. 1). Only 14 participants were lost to follow-up between t0 and t1 (conducted from March to November 2019), leading to 66 remaining matched pairs for the analysis. Gardeners came from 19 different community gardens and held either a collective (52.6%) or individual (47.4%) plot. We saw no difference with mixed models on gardeners using garden type as fixed effect (collective versus individual plots), therefore the data on garden type were pooled together. Garden-to-household distance varied between gardeners: 72,7% walked or biked to the garden (mean travel time: 8.6 min) and 27.3% used car or public transportation (mean travel time: 21.2 min).

### Participant characteristics

Sociodemographic characteristics of the participants at baseline are shown in Table 1. The mean age of gardeners was 44.0 years. Most of them were females, held a university degree and reported having no past experience in gardening. There were some differences between the gardeners and the controls at baseline: gardeners had lower education level, lower BMI and gardeners’ households reported lower percentages of meals consumed outside of the home.

**Table 1:** Baseline sociodemographic characteristics of gardeners and non-gardeners of the JArDinS study

	Gardeners (***n*** = 66)	Non-gardeners (***n*** = 66)	***P***-value^***a***^
**Matching criteria**			
*Individual level *			
Age (year), mean (SD):	44.0 (14.0)	44.9 (13.7)	0.706
Females, n (%)	50 (75.8)	50 (75.8)	0.808
*Household level*			
Household structure, n (%):			0.999
Single adult with no child	25 (37.9)	25 (37.9)	
Single adult with at least 1 child	6 (9.1)	7 (10.6)	
> 1 adult with no child	20 (30.3)	20 (30.3)	
> 1 adult with at least 1 child	15 (22.7)	14 (21.2)	
Household income (€/month/consumption unit), n (%):			0.605
< 1110	14 (21.2)	11 (16.7)	
1110–1999	29 (43.9)	26 (39.4)	
2000–2699	11 (16.7)	17 (25.8)	
≥ 2700	10 (15.2)	9 (13.6)	
NA	2 (3.0)	3 (4.5)	
** Other sociodemographic characteristics**			
* Individual level*			
Education level, n (%):			
Elementary school	1 (1.5)	1 (1.5)	**0.049**^**b**^
Secondary school	15 (22.7)	6 (9.1)	
University or equivalent	50 (75.8)	59 (89.4)	
BMI (kg/m^2^)	22.6 (3.0)	23.8 (4.0)	**0.046**
No meat or fish eater, n (%)	13 (19.7)	8 (12.1)	0.157
Experience in gardening, n (%):			1.000^**c**^
Beginner	47 (71.2)	47 (71.2)	
Intermediate	19 (28.8)	17 (25.8)	
Advanced	0	2 (3.0)	
* Household level*			
Percentage of meals consumed outside of the home in total household meals (%), mean (SD)	16.4 (11.7)	20.6 (15.3)	**0.033**

^a^ *P*-value for the difference between the two groups using paired t-test for age and BMI, and McNemar test for other variables

^b^ The first two categories were grouped together for statistical analysis

^c^ The last two categories were grouped together for statistical analysis

### Change in sustainability of lifestyles

Results of the mixed-effect models are shown in Table 2. At baseline, there were no pre-existing differences between the two groups on any of the components of lifestyles except for the contribution of added fats & seasonings to total household food expenditure. For both groups, physical activity significantly decreased between t0 and t1, while inactivity increased. Other changes due to time were, in both groups, an increase of BMI, an increase of sensitivity to food waste and a decrease in beverages expenditure share. At t1, half of gardeners (*n* = 32) had not retrieved any fruit or vegetables from the garden and for the others the mean quantity harvested was 38.9 (SD 44.1) g/d per person [median: 23.3, IQR: 3.6–55.9] (data not shown).

**Table 2:** Group differences and time effect of components of lifestyles sustainability among gardeners and non-gardeners^a^

Sustainability components, means (SD)^**b**^	Model^**c**^	Gardeners (*n* = 66)		Non-gardeners (*n* = 66)		Group *P*-value	Time *P*-value	Group* Time *P*-value
		t0	t1	t0	t1			
** Social/Health dimension **								
*Healthiness of household’s food supply*^d,e^								
Fruit & Vegetables^f^ (g/d/p)	B	402.4 (238.2)	400.0 (231.2)	433.6 (285.4)	445.6 (304.5)	0.241	0.637	0.999
MAR (% adequacy/2000 kcal)	B	76.5 (7.3)	75.8 (8.1)	76.3 (7.1)	76.9 (6.5)	0.679	0.936	0.356
MER (% excess/2000 kcal)	B	96.6 (19.5)	96.1 (23.4)	100.2 (25.3)	98.8 (29.7)	0.617	0.705	0.844
HPI [range: 0–15]	B	8.7 (2.1)	9.0 (2.1)	9.0 (2.3)	9.1 (1.9)	0.218	0.282	0.604
*Physical activity*^*g*^								
PAEE (kJ/kg/d)	A	43.2 (13.8)	40.3 (12.3)	41.9 (12.4)	39.9 (13.5)	0.489	**0.027**	0.664
Inactivity (h/d)	A	9.4 (1.4)	9.9 (1.5)	9.4 (1.5)	9.8 (1.4)	0.333	**< 0.0001**	0.995
Low-intensity activity (h/d)	A	2.8 (0.8)	2.7 (0.9)	2.8 (1.0)	2.6 (0.8)	0.792	**0.003**	0.544
Moderate-to-vigorous intensity activity (h/d)	A	1.9 (0.9)	1.6 (0.7)	1.8 (0.7)	1.7 (0.8)	0.555	**< 0.001**	0.362
BMI (kg/m^2^)	D	22.6 (3.1)	22.8 (3.1)	23.8 (4.0)	23.9 (4.1)	0.111	**0.038**	0.679
WEMWBS [range: 14–70]	C	51.1 (6.7)	51.5 (6.9)	51.8 (6.7)	51.5 (5.7)	0.406	0.899	0.546
UCLA Loneliness Scale [range: 20–80]	C	42.1 (10.4)	40.1 (10.9)	40.1 (9.8)	40.5 (9.5)	0.727	0.570	0.227
**Environmental dimension**								
High sensitivity to food waste, n (%)	C	30 (45.5)	40 (60.6)	27 (40.9)	30 (45.5)	0.274	**0.018**	0.214
Nature Relatedness Scale [range: 1–5]	C	4.1 (0.5)	4.1 (0.5)	3.9 (0.5)	4.0 (0.5)	0.060	0.198	0.395
*Environmental impact of household’s food supply*^d,e^								
GHGE (in g CO_2_eq/2000 kcal)^f^	B	3099 (997)	3151 (1131)	3294 (886)	3240 (889)	0.382	0.836	0.678
Atmospheric acidification (in g SO_2_eq/2000 kcal)^f^	B	33.1 (12.2)	33.3 (12.0)	37.6 (15.0)	35.4 (12.1)	0.256	0.398	0.373
Marine eutrophication (in g Neq/2000 kcal)^f^	B	11.9 (3)	12.5 (3.9)	13.3 (3.5)	13 (3.9)	0.124	0.972	0.271
Animal to plant protein ratio of household food supply^f^	B	56.9 (16.1)	56.4 (17.4)	61.8 (15.4)	59.1 (15.6)	0.368	0.091	0.245
**Economic dimension**								
Household food expenditure (€/d/p)^d,e^	B	7.0 (3.1)	6.7 (3.2)	6.8 (3.3)	6.8 (3.2)	0.841	0.682	0.630
*Expenditure share by food groups (%)*^d,e^								
Fruits & Vegetables	B	26.5 (11.1)	27.0 (10.4)	26.6 (12.3)	29.4 (15.6)	0.258	0.100	0.237
Starches	B	10.1 (5.2)	10.6 (5.1)	9.2 (4.7)	8.8 (4.5)	0.177	0.836	0.228
Meat, fish & Eggs	B	18.8 (9.5)	18.7 (10.2)	20.2 (9.2)	20.2 (10.9)	0.507	0.908	0.901
Dairy products	B	11.8 (5.1)	11.5 (4.8)	11.3 (4.4)	11.2 (5.2)	0.495	0.669	0.825
Mixed dishes^f^	B	8.9 (6.2)	8.4 (6.1)	8.3 (6.1)	8.9 (8.2)	0.098	0.496	0.998
Sweet products	B	10.4 (5.5)	11.7 (8.2)	11.1 (5.6)	10.1 (6.0)	0.853	0.855	0.078
Added fats & seasonings^f^	B	4.4 (3.0)	4.9 (2.8)	3.5 (2.6)	3.6 (2.6)	**0.003**	0.507	0.216
Beverages^f^	B	9.5 (6.2)	8.4 (5.8)	10.1 (7.4)	8.1 (6.1)	0.745	**0.021**	0.240

^a^ *Abbreviations*: *GHGE* GreenHouse Gas Emissions, *HPI* Healthy Purchase Index, *MAR* Mean Adequacy ratio, *MER* Mean Excess Ratio, *PAEE* Physical activity energy expenditure, *WEMBWS* The Warwick-Edinburgh Mental Wellbeing Scale

^b^ Unless specified

^c^ Model A was adjusted on BMI and education level. Model B = Model A + percentage of meals consumed outside of the home. Model C = Model A + social desirability scale. Model D was adjusted on education level, percentage of meals consumed outside of the home and social desirability scale

^d^ Variable measured at the household level and not at the individual one

^e^ Including produce from the garden and foods from gifts or food aid. For food expenditure variables, a mean price was attributed to these foods (see Method section)

^f^ Variable was log-transformed to improve normality

^g^ Participants with less than 3 valid days (≥ 10 h of wearing the accelerometer wearing during daytime) were excluded from the analysis resulting in 65 gardeners and 65 controls at t0, and 64 gardeners and 62 controls at t1

No significant impact of participating in a community garden was observed on the main outcome variable (household fruit and vegetables supply) or on any of the other outcomes, as shown by the lack of significance for the interaction term (group*time) (Table 2). Even when removing the theoretical expenditure attributed to produce from the garden, there was no measurable impact of garden participation on total food expenditure and on food-group expenditure shares (data not shown).

**Fig. 2:**
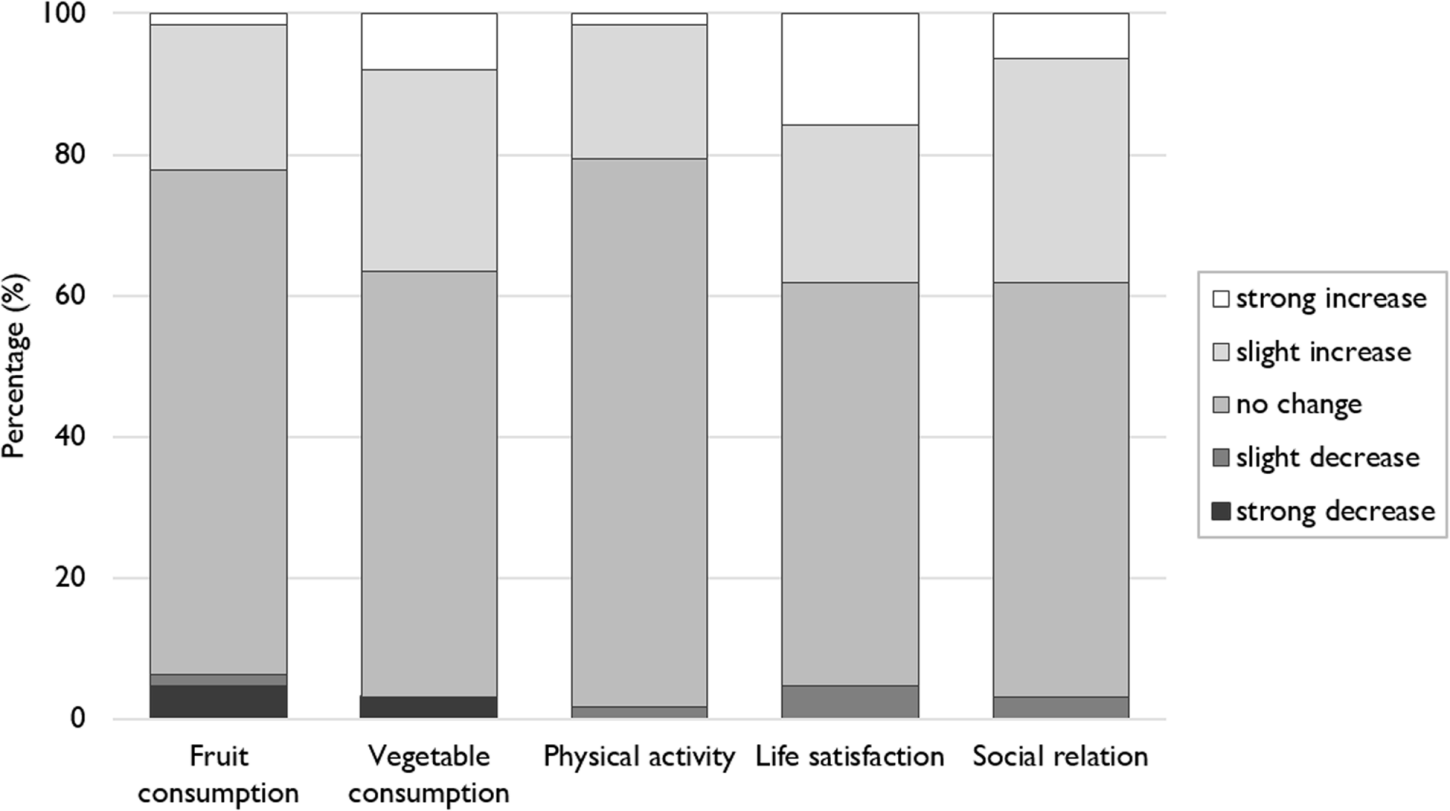
Perceived change by gardeners in fruit consumption, vegetable consumption, physical activity, life satisfaction and social relation after the first year in a community garden (*n* = 63)

At t1, 24% of the gardeners surveyed had dropped out the garden during the year. There was inter-individual variability in the frequency of garden attendance during the year, the majority of gardeners visiting the garden at least once a month throughout the year (56.1%) or over a period from 6 to 9 months (18.2%), while others visited the garden for shorter periods, from 3 to 6 months (16.6%) or only few times a year (9.1%). Post-hoc analyses also showed a non-significant effect of participating in a community garden on outcome variables on sub-samples including active gardeners only (*n* = 37) or those who did not drop out the garden during the year (*n* = 50) (Additional files 3 and 4). In the post-survey questionnaire, the majority of gardeners stated they did not perceive any change in their fruit and vegetable consumption, physical activity, life satisfaction and social relation due to gardening (Fig. 2).

### Qualitative evaluation

The qualitative evaluation provided some plausible explanations for the lack of change in gardeners’ lifestyles observed in the quantitative study. Of the 15 gardeners interviewed, 9 were active gardeners, 4 visited the garden over a period from 6 to 9 months and 2 less than 3 months.

Nine respondents perceived no change in their lifestyles after 1 year of gardening. This lack of perceived benefit cannot only be explained by having a negative gardening experience, as one gardener described: “My life is the same as before [the garden], but it’s true that it’s a plus to have this garden anyway. It’s one of the positive things in my life, but after that it’s not really changing my life actually” (Female 1, age 54). A couple of respondents pointed out that participation in the garden was just part of their health and environmental consciousness: “I was already a nature enthusiast. What I mean is, that I have always been environmentally conscious, and caring about nature” (Male 1, age 34). It is also possible that a follow-up period of 1 year was not sufficient to perceive changes. For example, one woman shared: “I hope I’ll improve over time” (Female 6, age 63).

The interview also highlighted several barriers to community garden participation, the most frequently mentioned being the lack of time to garden. Nine gardeners, mostly females, confessed facing greater challenges balancing the demands of gardening with their personal and professional lives, which could lead to feelings of guilt. One gardener mentioned:

At first, I didn’t know if I would have enough time to invest in the garden […]. Indeed, it actually requires a lot of time. […] You see what time I get home from work and everything. I don’t have time for this. I see them working. At first, I felt guilty, I thought, “Oh, now I see them and so on, and then I don’t go there” I had no experience of gardening and how long it took. […] It’s true that it's great, it’s almost in a meditative state, it brings me a lot of well-being, but it takes a lot of time (Female 3, age 42).

The demands of daily life are an important factor associated with participation, regardless of gardeners’ motivation:

I struggle to go there regularly. I said yes, I signed up, and I thought I was going to participate a lot. In the end, not that much, because I had a lot of things happen. My place of work changed. My husband was very ill. My father was very ill, while he’s in Toulouse, etc. My car is broken, there are things like that... […] If it was in the same neighbourhood it would be easier. (Female 8, age 61).

The distance between the garden and the house can be an additional source of discouragement outweighing benefits from the garden:

What’s difficult is not the time, it is the trip. It’s hard to organize because you don’t come for 10 minutes, you come to stay longer than that. Therefore, the travel time, and the round trips make it challenging to always find the time to do it. If it was just next door, it would have been easier. (Female 4, age 41).

These quotations underline how difficult it was for participants to make time in their busy lives to access the garden away from their homes. Other barriers mentioned by the gardeners were difficulties of gardening and the lack of experience (*n* = 3). One gardener explained that:

What I first noticed was that this work is not actually simple as people think. It’s not just planting and harvesting. You have to know what you’re planting. You have to know many plants. I know some of them. No, it’s not an easy job. You need to also know how to conserve water because you can garden by using lot of water especially here in the South. It's a bit risky. It’s depends. Since we don't want to use pesticides, we want it to be as natural as possible. It’s not always an easy job. (Female 7, age 65).

Lack of gardening knowledge, especially for beginning gardeners, can lead to a negative gardening experience if they are not helped or supervised by other more expert gardeners, as explained by this woman:

I had to make mistakes, I don't know which ones because we don't have-- It's a garden where there aren't many people, so it's hard to get advice, it's hard to look at on the internet. That's not what I expect from a garden, it’s the discussion, that's what I want to get tips, because there are people who have been doing this for a longer time. It didn't work for me. (Female 2, age 60).

The difficulty of gardening, especially soil preparation and bending down can be a hindrance for people with fragile health such as the elderly: “After all, I’m 63 years old, and I don’t have the strength, the physical resistance to mix the soil. Moreover, I sleep very badly because I feel pain everywhere. (Female 6, age 63).

Although many gardeners mention the friendly moments in the garden, tension related to the management of garden can arise and lead to conflicts between gardeners. This was notably the case for one of the interviewed gardeners:

I refuse all fixed patterns of thinking. These people, unfortunately, behind their participatory democracy side, are oligarchs, sorry. I can't stand the oligarchy. Oligarchy is the power of a few behind a pseudo-democratic form. I don't want to be given orders, at least not in the garden, so I refuse to see them and leave. (Female 10, age 48).

## Discussion

JArDinS is the first longitudinal study to examine the causal effect on gardeners’ lifestyles of participating in a community garden, 1 year after entry. We found no impact of one-year garden participation on healthiness of household’s food supply, physical activity, BMI, mental well-being and social health, connection to nature, sensibility to food waste, as well as, environmental impact and expenditure of food supply. The present results are not in line with previous studies that concluded on the health, social, environmental and economic benefits of community gardening [11–13, 15–17, 19, 20, 30]. The JArDinS study was specifically implemented to overcome shortcomings from the existing literature. Our results suggest that previous cross-sectional studies have been subject to selection bias. Indeed, it is likely those studies have been conducted on a small percentage of highly motivated and experienced gardeners who did not abandon the activity because they already had positive health consciousness and/or environmental attitudes. Additionally, previous studies were conducted predominantly amongst vulnerable populations such as socioeconomically disadvantaged neighborhoods or ethnic minorities in U.S. cities [45, 46], and therefore outcomes cannot easily be generalized to other settings and other populations. Lastly, in previous studies, health outcomes were mostly collected by declarative questionnaires, which are subject to desirability and memory bias [47]. In particular, frequency of fruit and vegetable intake was mainly estimated based on short food frequency questionnaire [11–15], which validity is moderate [48]. The measure of household food and beverage purchasing behaviour through the collection of food receipts and records offer a more objective approach to estimate dietary behaviours at the household level [49]. Similarly, self-reported physical activity is less accurate than objective methods for estimating PAEE and PA intensity [50]. Among the objective measurement tools, accelerometers allow to capture large amounts of data over several days and have gained popularity to quantify more precisely PAEE and PA intensity [51].

Our results suggest that practicing gardening for 1 year in a community garden may not be sufficient to modify health and sustainability behaviors in a French setting. The observed low gardener involvement might explain the lack of effect of community gardening on gardeners’ lifestyles. However, post-hoc analysis on active gardeners only did not show a positive effect of gardening on gardeners’ lifestyles, but these findings are to be taken with caution because the reduction of the sample also reduces statistical power. Furthermore, it can also be argued that even the most active gardeners did not visit the garden enough to result in a lifestyle change. In the litterature, strong evidence of the health benefits of gardening mainly comes from gardening programs in school or medical settings where the activity is supervised and carried out regularly, usually on a weekly basis [3, 52, 53]. Additionally, in these institutionalized settings the gardening activity is often accompanied by other actions such as, in school, nutrition education courses, cooking classes, introduction of healthy foods in the school lunchrooms [3]. Hence, multicomponent programs are more likely to modify health outcomes [3]. More broadly, many questions regarding the health benefits of exposure to green spaces remain unanswered and further studies are needed to understand which forms of nature contact are the most beneficial, the duration of exposure needed, the variation of effects across populations and settings as well as the psychological pathways involved [54].

Interviews showed that lack of time and knowledge about gardening, health problems or conflicts with other gardeners contributed to gardener discouragement. While motivations for participating in community gardens are well documented in the literature, there are few studies that focus on the barriers encountered by gardeners and they all agree that lack of time, knowledge, practical skills, physical capacity or conflicting personal and social expectations are major hindrances to gardener involvement [55–59]. These obstacles can be expected to be particularly difficult for beginner gardeners; thus preventing them from changing their behaviour. Additionally, it is likely that French gardeners entered the gardens with a higher quality diet and more physical activity compared to gardeners in low-income areas of the U.S. more inclined to suffer from inequalities in access to healthy food and recreational facilities [60, 61]. The hypothesis of a pre-established health consciousness of gardeners before entering the garden was supported by the qualitative evaluation. Some gardeners also expressed pre-existing environmental awareness. We found that, compared to non-gardeners, only active gardeners had a slightly but significantly higher connection to nature at baseline. While there was no difference between less active gardeners and non-gardeners, this significant trend reinforces the idea that the most involved gardeners might have been more predisposed to stay in the garden because of positive attitudes enabling them to better cope with the difficulties of gardening.

Given that community garden participation is particularly likely to enhance health and wellbeing of vulnerable populations [46], one can assume that the recruitment of less educated, low-income gardeners would have led to different conclusions. Nevertheless, a survey conducted in Toronto revealed that food-insecure households considered gardening programs unsuited to their busy schedules, interests, or needs and preferred to use food banks instead [58, 62]. An ongoing trial using rigorous assessment methods (24 h recall and accelerometer) in Denver (USA) will provide further insight regarding the causal relationship between community gardening and health in a mixed-income population in a non-European setting [23].

Surprisingly, we found an increase in inactivity and BMI after 1 year in both groups. It is likely that age-related weight gain explained the BMI increase. Participants gained on average 0.4 kg in the year, which is similar to weight change observed in three U.S. cohorts [63]. Regarding inactivity, participants were used to the Actigraph at t1 and probably forgot that they were wearing it, resulting in a decrease of the desire to emphasize their “healthy behavior”.

We acknowledge that our study is not without limitations. First, the non-random design conveys an important risk of bias [64]. One major threat of is the risk of non-comparability between groups [65]. At baseline gardeners had slightly lower level of education, lower BMI and their households consumed less meals outside of the home, which is consistent with their higher expenditure for added fats & seasonings (i.e. cooking ingredients) than non-gardeners. Random sampling was not achievable in our setting because new membership and plot renewal in community gardens are under the control of local authorities or private managers. Nevertheless, longitudinal pre-post quasi-experimental designs offer robust alternative to randomized control trials to determine a causal relationship [66]. In addition, we used a pairwise matching process controlling for individual-level and contextual-level variables to reduce selection bias and strengthen internal validity of the study [66]. Furthermore natural experiments improve external validity by giving a more realistic representation of the effectiveness of an intervention in a real world setting [66]. Second, despite investigating several components of lifestyles across the three dimensions of sustainability, it is always possible that other unmeasured variables might have changed following gardening participation. Third, the diversity of the data collection tools and the longitudinal follow-up required a significant involvement of the participants which could have led to the recruitment of people more concerned about their health than the general population, and therefore underestimate the effect of the intervention [67]. In our case, the majority of participants held a university degree and displayed healthier dietary patterns at baseline than the French general population [68], with a higher intake of fruits, vegetables and nuts (398 vs 264 g/d per person) (data not shown). Fourth, it can also be argued that insufficient statistical power and inability of the data collection tools to detect change could explain the lack of results, nevertheless, the sample was large enough to observe significant differences between the groups initially, and findings from the post-survey questionnaire support the conclusion that no changes occurred for the majority of gardeners during the year. Fifth, to avoid overburdening participants, we decided not to record dining out food purchase data. Nevertheless, despite shifts in eating patterns favouring eating out, French food consumption is still mainly driven by household food purchases [69]. Sixth, knowing that changes in diet take time, a follow-up longer than 1 year might be needed to detect a change in food practices. However, given the high turnover in Montpellier gardens (gardeners quitting, moving, etc.), a longer follow-up would have resulted in important attrition, selection bias and loss of statistical power.

## Conclusions

Our study did not find a positive impact of participation in a community garden on the sustainability of lifestyles. The qualitative evaluation identified difficulties encountered by the gardeners such as lack of time and insufficient gardening knowledge, as well as health problems and conflicts with other gardeners, thereby providing leads to identify solutions to overcome these barriers. At a time when many cities are planning to establish community gardens on their territories, our findings call on public authorities and gardening leaders to rethink the management and organisation of gardens. In light of our results, the establishment of gardens in the immediate vicinity of housing could facilitate a more regular use of the gardens. The presence of facilitators who supervise the garden, assist gardeners, promote group dynamics and serve as a mediator in conflict management could also favour the integration and long-term participation of individuals with a variety of cultural and socio-economic profiles. An evaluation of these new gardening formats through future intervention studies may provide evidence of the relevance of using community gardens to accompany the urban population towards the adoption of more sustainable lifestyles.

## Supplementary Information

**Additional file 1.** Data analyses of food supply diary and accelerometer.

**Additional file 2. **Additional information from the online questionnaire of the *JArDinS* study.

**Additional file 3. **Group differences and time effect of lifestyles components among active gardeners (≥ 1 visit per month throughout the year, *n* = 37) and paired non-gardeners.

**Additional file 4. **Group differences and time effect of lifestyles components of gardeners who did not dropped out the garden during the year (*n* = 50) and paired non-gardeners.

## Data Availability

The data that support the findings of this study are available from MT but restrictions apply to the availability of these data, which were used under license for the current study, and so are not publicly available. Data are however available from the authors upon reasonable request and with permission of data controller (CM) and interested parties.
