# Correction to: Improving lifestyles sustainability through community gardening: results and lessons learnt from the JArDinS quasi-experimental study

**DOI:** 10.1186/s12889-020-10010-1

**Published:** 2021-01-04

**Authors:** Marion Tharrey, Ashby Sachs, Marlène Perignon, Chantal Simon, Caroline Mejean, Jill Litt, Nicole Darmon

**Affiliations:** 1 MOISA, Univ Montpellier, CIRAD, CIHEAM-IAMM, INRAE, Institut Agro, Montpellier, France; 2 University of Colorado Boulder, Boulder, CO USA; 3 CarMen Laboratory, INSERM 1060, INRA 1397, University of Lyon, F-69600 Oullins, France; 4 ISGlobal, Barcelona, Spain

**Correction to: BMC Public Health 20, 1798 (2020)**

**https://doi.org/10.1186/s12889-020-09836-6**

It was highlighted that the original article [1] contained some incorrect statements. This Correction article shows the incorrect and correct version of the sentences. The original article has been updated.

**Incorrect**

Gardeners came from 19 different community gardens and held either a collective (52.6%) or individual (47.4%) plot.

**Correct**

Gardeners came from 19 different community gardens and held either a collective (68.2%) or individual (31.8%) plot.

**Incorrect**

At t1, half of gardeners (*n* = 32) had not retrieved any fruit or vegetables from the garden and for the others the mean quantity harvested was 38.9 (SD 44.1) g/d per person [median: 23.3, IQR: 3.6-55.9] (data not shown)."

**Correct**

At t1, more than half of gardeners (*n* = 38) had not retrieved any fruit or vegetables from the garden and for the others the mean quantity harvested was 33.7 (SD 40.7) g/d per person [median: 17.1, IQR: 3.6-49.9] (data not shown).

**Incorrect**

and animal to plant protein ratio of household food supply;

**Correct**

and contribution of animal protein to total protein of household food supply;

**Incorrect** (Table 3)

Animal to plant protein ratio of household food supply

**Correct**

Animal protein (in % of total protein)
