# Supplementary material for: Improving lifestyles sustainability through community gardening: results and lessons learnt from the JArDinS quasi-experimental study
Source: BMC Public Health. 2020 Nov 26;20:1798. doi: 10.1186/s12889-020-09836-6 (PMC7690132; doi:10.1186/s12889-020-09836-6)
Supplement: Supplementary file 1 — Additional file 1. Data analyses of food supply diary and accelerometer. [file 12889_2020_9836_MOESM1_ESM.docx]

**Additional file 1.** Data analyses of food supply diary and accelerometer

**Food supply diary**

*Healthiness of household’s food supply*. The food diary supply provided a detailed record of foods (including purchases, donation and garden produce) entering the household over 1-month period. A database of monthly food purchases was then created by compiling information on date of purchase, quantity and price of all food items purchased. Food, energy and nutrient content of household monthly purchases were estimated following a previously described procedure (1). A French food nutrient composition database completed by newly consumed foods and recipes provided was used to determine the nutrient content of all the foods, as consumed (i.e., after peeling, boning, water loss or gain during cooking, etc.) (2). The primary outcome measure of healthiness was the household fruit and vegetables supply (in g/d per person) calculated as the sum of all fruit and vegetables recorded (in g as consumed) during the period of data collection, divided by the number of days of data collection and the number of household members. We also estimated the nutritional quality of household’s food supply using two indicators of nutritional quality : the mean adequacy ratio (MAR) which an indicator of good nutritional quality and represents the mean percentage of daily recommended intakes for 23 key nutrients (3), and the mean excess ratio (MER), which is an indicator of bad nutritional quality and represents the mean percentage of daily maximum recommended values for 3 harmful nutrients, namely saturated fatty acids, sodium, and free sugars (4). The MAR and the MER were calculated for 2000 kcal of household food supply. We also calculated the HPI (Healthy Purchase Index), an index estimating the healthiness of household food purchases, based on food expenditure only (5). Total scores range of HPI from 0 to 15, with higher scores indicating healthier household food purchases.

*Environmental impact of household’s food supply*. The GHGE (in g CO_2_eq), atmospheric acidification (in g SO_2_eq) and marine eutrophication (in g Neq) associated with household food purchases were estimated based on the environmental impacts of each food item. An environmental food database providing the GHGE, acidification and eutrophication estimates (per 100 g of edible part) for 541 food items was used. The estimates were assigned based on an hybrid method combining input/output and life-cycle assessment approaches and reflecting food products as consumed in the French market, as previously described (6). Each food item from the food supply database that was present in the list of 541 food items from the environmental database was attributed the corresponding environmental estimates. For the other food purchased, environmental estimates were allocated by linking each of them to the “nutritionally” closest food among the 541 food items. To do so, we calculated the nutritional Euclidean distance between food items within each food category based on their energy, water, protein, fiber, alcohol, sodium, calcium, iron, zinc, vitamin A, vitamin E, vitamin C, fats and saturated fatty acid contents. We also estimate the animal to plant protein ratio of household food supply as the ratio of protein from animal-sourced foods to protein from plants.

*Household food expenditure and expenditure share by food groups*. The expenditure for each food item purchased during the month of data collection was summed to calculate total household food expenditure. A theoretical price using the mean observed food price was assigned to produce picked from the garden, and to foods freely received from family, friends, colleagues or food aid organizations. Missing information on the price of a food was estimated by multiplying the weight of the food by the observed mean price per kilogram of that food item.

**Physical activity**

Participants were instructed to wear a triaxial accelerometer (wGT3X-BT or wActiSleep-BT, Actigraph, Pensacola,FL, USA) for 9 consecutive days, 24h a day at the right hip. They were asked to record in an activity logbook the time they went to and get out of bed each night, as well the time window and reason/type of any device removal and exercise. Raw acceleration and counts per minute were downloaded using the manufacturer’s software (ActiLife version 6.13). Sleep time was identified from counts per minutes as previously described (7) and daytime non-wear periods determined as sequences of 20 minutes with no significant raw acceleration. Participants with less than 3 valid days (defined as at least 10h wearing during daytime) were excluded from the analysis. Physical activity energy expenditure (PAEE) and time spent during daytime in activities of different intensities were using a previously validated model that combines an automatic activity-recognition algorithm with an activity-specific count-based model (8)*.* Activities were classified as inactivity (< 1.5 METs), light intensity activities (between 1.5 and 3 METs) and moderate-to-very vigorous intensity activities (> 3 METs).

**References**

1. Marty L, Dubois C, Gaubard MS, Maidon A, Lesturgeon A, Gaigi H, et al. Higher nutritional quality at no additional cost among low-income households: insights from food purchases of “positive deviants.” Am J Clin Nutr. 2015;102:190–8.

2. ANSES. Ciqual Table de composition nutritionnelle des aliments. Internet : https://ciqual.anses.fr/#/cms/telechargement/node/20. Accessed 3 Apr 2020.

3. Maillot M, Darmon N, Vieux F, Drewnowski A. Low energy density and high nutritional quality are each associated with higher diet costs in French adults. Am J Clin Nutr. 2007;86:690–6.

4. Vieux F, Soler L-G, Touazi D, Darmon N. High nutritional quality is not associated with low greenhouse gas emissions in self-selected diets of French adults. Am J Clin Nutr. 2013;97:569–83.

5. Tharrey M, Dubois C, Maillot M, Vieux F, Méjean C, Perignon M, et al. Development of the Healthy Purchase Index (HPI): a scoring system to assess the nutritional quality of household food purchases. Public Health Nutr. 2019;22:765–75.

6. Bertoluci G, Masset G, Gomy C, Mottet J, Darmon N. How to Build a Standardized Country-Specific Environmental Food Database for Nutritional Epidemiology Studies. PLoS One. 2016;11:e0150617.

7. Barreira T V., Schuna JM, Mire EF, Katzmarzyk PT, Chaput JP, Leduc G, et al. Identifying children’s nocturnal sleep using 24-h waist accelerometry. Med Sci Sports Exerc. 2015;47:937–43.

8. Garnotel M, Bastian T, Romero-Ugalde H-M, Maire A, Dugas J, Zahariev A, et al. Prior automatic posture and activity identification improves physical activity energy expenditure prediction from hip-worn triaxial accelerometry. J Appl Physiol. 2018;124:780–90.
