# Supplementary material for: Improving lifestyles sustainability through community gardening: results and lessons learnt from the JArDinS quasi-experimental study
Source: BMC Public Health. 2020 Nov 26;20:1798. doi: 10.1186/s12889-020-09836-6 (PMC7690132; doi:10.1186/s12889-020-09836-6)
Supplement: Supplementary file 2 — Additional file 2. Additional information from the online questionnaire of the JArDinS study. [file 12889_2020_9836_MOESM2_ESM.docx]

**Additional file 2.** Additional information from the online questionnaire of the *JArDinS* study

**Part 1: Your personal data**

A1. First name: .................................

A2. Gender: 🞎 Male 🞎 Female

A3. Date of birth: ............./............/................

A4. Country of birth: .................................................................

A5. Phone number (main): .................................................................

A6. Phone number (secondary): .................................................................

A7. E-mail: .................................................................

A8. Please confirm your e-mail: .................................................................

A9. What is your weight? ................................kg

A10. What is your height? ................................cm

A11. What is the highest degree you have obtained?

🞎 No diploma, primary school certificate, general certificate of secondary education (GCSE) or equivalent

🞎 Youth Training (NVQ Level 1,2), BTEC First Diploma or equivalent

🞎 Bachelor's degree, BTEC National Diploma or equivalent

🞎 Baccalaureate +2 years (BTEC Higher National Diploma, Diploma of Higher Education or equivalent)

🞎 Baccalaureate +3 or +4 years (BA, BS/BSc, MS/MSc, MA)

🞎 Baccalaureate +5 years or more

A12. Are you the main person in charge of grocery shopping for your household?

🞎 Yes, I am the main person in charge of the household grocery shopping.

🞎 Yes and no, it depends on what type of grocery shopping and my schedule.

🞎 No, I do very little grocery shopping.

A13. Home address:

N° and street name: ...........................................................................................................

Additional address: ............................................................................................................

Postal code: ............................................................................................................

City, village, town: ............................................................................................................

A14. How many people (adults and children) regularly live at home with you? *Including children with shared custody*

………………………………………...............................

A15. How many of them are aged:

- 3 years old or less? ...............................

- between 4 and 13 years old (inclusive)? ...............................

- between 14 and 17 years old (inclusive)? ...............................

- 18 years or older (including yourself)? ...............................

A16. From the list below, check the box that corresponds to your household's net income before taxes^1,2^

| BY MONTH | Check the box | BY YEAR |
| --- | --- | --- |
| less than 600 euros |  | less than 7,200 euros |
| from 600 to less than 1,110 euros |  | from 7,200 to less than 13,300 euros |
| from 1,110 to less than 1,430 euros |  | from 13,300 to less than 17,170 euros |
| from 1,430 to less than 1,670 euros |  | from 17,170 to less than 20,040 euros |
| from 1,670 to less than 2,000 euros |  | from 20,040 to less than 24,050 euros |
| from 2,000 to less than 2,330 euros |  | from 24,050 to less than 28,000 euros |
| from 2,330 to less than 2,700 euros |  | from 28,000 to less than 32,290 euros |
| from 2,700 to less than 3,130 euros |  | from 32,290 to less than 37,510 euros |
| from 3,130 to less than 3,780 euros |  | from 37,510 to less than 45,400 euros |
| from 3,780 to less than 4,800 euros |  | from 45,400 to less than 57,550 euros |
| 4,800 to less than 8,710 euros |  | 57,550 to less than 104,550 euros |
| plus de 8 710 euros |  | from 13,300 to less than 17,170 euros |
| Don't know |  |  |
| Does not wish to answer |  |  |

*^1^ corresponds to the sum of the resources declared on the income tax return, before any allowance*

*^2^ If have roommates, please do not take into account their income.*

A17. What is your current employment status?

🞎 Employed (full-time or part-time)^1^

🞎 Unemployed

🞎 Student^2^

🞎 Retired

🞎 At home

🞎 On disability / long term illness

🞎 In training

🞎 Other, (specify): .................................................................................

*^1^ Includes any person who claims to be working, even on a part-time or interim basis, whether as an employee, self-employed, employer or helper in the family business or operation, paid trainee, or member of the military contingent.*

*^2^ Includes any person whose main activity is to study at an educational institution. If you work more than 480 hours per year (or more than 40 hours per month) while you are studying, please check the box "employed". Otherwise, check the "student" box.*

A18. This question is about your consumption of some food groups. Please check the answer that you feel is most accurate.

Generally, you eat the products below:

|  | Rather at home | Rather out of home | As much at home than outside the home | I don’t eat them^1^ |
| --- | --- | --- | --- | --- |
| Meat |  |  |  |  |
| Fish |  |  |  |  |
| Fruit and vegetables |  |  |  |  |
| Dairy products |  |  |  |  |

*^1^ or in a very exceptional way because I am vegetarian/vegan, I don't like them or I can't for health reasons*

A19. This question concerns food consumption outside your home. Please check the answer that you feel is most accurate.

Generally, you go to the places below:

|  | Every day | 4-6 times/  week | 1-3 times/  week | 1-3 times/  month | < 1 time/  month | Never or exceptionally |
| --- | --- | --- | --- | --- | --- | --- |
| Canteen or company restaurant |  |  |  |  |  |  |
| Restaurant and caterer (eaten on site or taken **away** **from home**) |  |  |  |  |  |  |
| Fast-food^1^ (eaten on site or taken **away** **from home**) |  |  |  |  |  |  |
| At a relative's home (family, friend, etc...) |  |  |  |  |  |  |

*^1^ Restaurants distributing for a low price and at any time products prepared quickly and ready to eat such as hamburgers, sandwiches, fries, hotdogs, tacos...*

**Part 2: Your gardening experience**

B1. Have you ever gardened?

🞎 Yes

🞎 No *🡪 Go directly to Part 3.*

B2. If yes, please check the proposal(s) that best suit(s) you among the following sentences (several possible answers):

🞎 I have gardened with my family and friends as a child but I never had a vegetable garden.

🞎 I grow or have grown a few vegetables and/or aromatic plants on my balcony/terrace.

🞎 I have been gardening for several years in a garden (mine or a relative's) but I was forced to stop (moving, health problem...).

🞎 I have been gardening for several years in a garden (mine or a relative's) and I still continue to do so more or less regularly.

🞎 I tried to grow a few plants without really gardening (I didn't have time / I didn't like it too much / I didn't have a green thumb...).

B3. You consider yourself as a:

🞎 Beginner gardener

🞎 Intermediate gardener

🞎 Confirmed gardener

**Part 3 (for gardeners only at baseline): The community garden**

C1. What is the name of the community garden you are going to?

…………………………………

C2. Do you have (/will you have) an individual or collective plot?

🞎 Individual plot

🞎 Collective plot

**Part 3 (for gardeners at year 1): The community garden**

C1. Were there periods when you did not go to the community garden for more than 1 month in a row? If yes, please specify the date and duration. *Ex: I did not go to the garden for 3 months, from January to March 2019.*

…………………..…………………..…………………..…………………..…………………..……….

C2. Apart from these periods, how often did you go to the garden during the past year?

🞎 Once a week or more

🞎 1 to 3 times a month

🞎 Less than once a month

C3. How long did it take you to get to the garden? ……………………………….

C4. What means of transportation do you primarily use to get to the garden?

🞎 On foot

🞎 Bicycle

🞎 Bus

🞎 Tramway

🞎 Motorcycle/scooter

🞎 Car

**Part 4: Other household members**

**During the first visit of the investigator you received a household food supply diary to collect information on all foods and beverages entering the household during the coming month (including purchases, donation and garden produce).**

D1. How many people living in your home over the next month will be concerned by these food supplies *(consider only those who live in the home for at least 7 consecutive or non-consecutive days):*

……………………………….. people

D2. How many of them are aged:

- 3 years old or less? ...............................

- between 4 and 13 years old (inclusive)? ...............................

- between 14 and 17 years old (inclusive)? ...............................

- 18 years or older (including yourself)? ...............................

**For each person concerned by the household food supply, please provide the following information.** (You can reconnect later and wait until the person are with you to finish filling out this part of the questionnaire)

**ADULTE n°X**

*(to be declined according to the number of adults)*

AX1. First name: …………………………….

AX2. Gender: 🞎 Male 🞎 Female

AX3. Date of birth: ......./......./........

AX4. Weight: ..........................kg

AX5. Height: ..........................cm

AX6. Were you at home during the month of data collection in the Food supply diary?

🞎 Yes

🞎 No. If not, estimate the number of days you were away: ...... days

AX7. What is your current employment status?

🞎 Employed (full-time or part-time)^1^

🞎 Unemployed

🞎 Student^2^

🞎 Retired

🞎 At home

🞎 On disability / long term illness

🞎 In training

🞎 Other, (specify): .................................................................................

*^1^ Includes any person who claims to be working, even on a part-time or interim basis, whether as an employee, self-employed, employer or helper in the family business or operation, paid trainee, or member of the military contingent.*

*^2^ Includes any person whose main activity is to study at an educational institution. If you work more than 480 hours per year (or more than 40 hours per month) while you are studying, please check the box "employed". Otherwise, check the "student" box.*

AX8. What is the highest degree you have obtained?

🞎 No diploma, primary school certificate, general certificate of secondary education » (GCSE) or equivalent

🞎 Youth Training (NVQ Level 1,2), BTEC First Diploma or equivalent

🞎 Bachelor's degree, BTEC National Diploma or equivalent

🞎 Baccalaureate +2 years (BTEC Higher National Diploma, Diploma of Higher Education or equivalent)

🞎 Baccalaureate +3 or +4 years (BA, BS/BSc, MS/MSc, MA)

🞎 Baccalaureate +5 years or more

AX9. This question is about your consumption of some food groups. Please check the answer that you feel is most accurate.

Generally, you eat the products below:

|  | Rather at home | Rather out of home | As much at home than outside the home | I don’t eat them^1^ |
| --- | --- | --- | --- | --- |
| Meat |  |  |  |  |
| Fish |  |  |  |  |
| Fruit and vegetables |  |  |  |  |
| Dairy products |  |  |  |  |

*^1^ or in a very exceptional way because I am vegetarian/vegan, I don't like them or I can't for health reasons*

AX10. This question concerns food consumption outside your home. Please check the answer that you feel is most accurate.

Generally, you go to the places below:

|  | Every day | 4-6 times/  week | 1-3 times/  week | 1-3 times/  month | < 1 time/  month | Never or exceptionally |
| --- | --- | --- | --- | --- | --- | --- |
| Canteen or company restaurant |  |  |  |  |  |  |
| Restaurant and caterer (eaten on site or taken **away** **from home**) |  |  |  |  |  |  |
| Fast-food^1^ (eaten on site or taken **away** **from home**) |  |  |  |  |  |  |
| At a relative's home (family, friend, etc...) |  |  |  |  |  |  |

*^1^ Restaurants distributing for a low price and at any time products prepared quickly and ready to eat such as hamburgers, sandwiches, fries, hotdogs, tacos...*

**CHILD n°X**

*(to be declined according to the number of adults)*

EX1. Sex: 🞎 Boy 🞎 Girl

EX2. Age: .................

EX3. Weight: ..........................kg

EX4. Height: ..........................cm

EX5. Was the child at your home during the month of data collection in the Food supply diary?

🞎 Yes

🞎 No. If not, estimate the number of days you were away: .......... days

EX6. This question is about the consumption of your child for some food groups. Please check the answer that you feel is most accurate.

Generally, your child eats the products below:

|  | Rather at home | Rather out of home | As much at home than outside the home | He/she doesn’t eat them^1^ |
| --- | --- | --- | --- | --- |
| Meat |  |  |  |  |
| Fish |  |  |  |  |
| Fruit and vegetables |  |  |  |  |
| Dairy products |  |  |  |  |

*^1^ or in a very exceptional way because he/she is vegetarian/vegan, doesn't like them or can't eat them for health reasons*

EX7. This question concerns the food consumption of your child outside your home. Please check the answer that you feel is most accurate.

Generally, your child goes to the places below:

|  | Every day | 4-6 times/  week | 1-3 times/  week | 1-3 times/  month | < 1 time/  month | Never or exceptionally |
| --- | --- | --- | --- | --- | --- | --- |
| Canteen or nursery |  |  |  |  |  |  |
| Restaurant and caterer (eaten on site or taken **away** **from home**) |  |  |  |  |  |  |
| Fast-food^1^ (eaten on site or taken **away** **from home**) |  |  |  |  |  |  |
| At a relative's home (family, friend, etc...) |  |  |  |  |  |  |

*^1^ Restaurants distributing for a low price and at any time products prepared quickly and ready to eat such as hamburgers, sandwiches, fries, hotdogs, tacos...*

**Post-survey questionnaire**

**Part 1: The garden's crops**

A1. During the gardening season, how much of the fruit intake of your household came from garden crops?

🞎 All the fruit came from the shared garden

🞎 More than half of the fruit came from the shared garden

🞎 A little less than half of the fruit came from the shared garden

🞎 A small part of the fruit came from the shared garden

🞎 No fruit came from the shared garden

A2. During the gardening season, how much of the vegetable intake of your household came from garden crops?

🞎 All the vegetable came from the shared garden

🞎 More than half of the vegetable came from the shared garden

🞎 A little less than half of the vegetable came from the shared garden

🞎 A small part of the vegetable came from the shared garden

🞎 No vegetable came from the shared garden

.

**Part 2: Perceived changed of lifestyles in the past year**

E2a. Compared to when you started the garden (more than a year ago now), would you say that the amount of fruit consumed in your home has changed?

🞎 Strongly increased

🞎 Slightly increased

🞎 Did not changed

🞎 Slightly decreased

🞎 Strongly decreased

E2b. Would you say this change is related to your participation in the garden?

🞎 Yes

🞎 Yes, in part

🞎 No

E3a. Compared to when you started the garden (more than a year ago now), would you say that the amount of vegetable consumed in your home has changed?

🞎 Strongly increased

🞎 Slightly increased

🞎 Did not changed

🞎 Slightly decreased

🞎 Strongly decreased

E3b. Would you say this change is related to your participation in the garden?

🞎 Yes

🞎 Yes, in part

🞎 No

E4a. Compared to when you started the garden (more than a year ago now), would you say that your physical activity has changed?

🞎 Strongly increased

🞎 Slightly increased

🞎 Did not changed

🞎 Slightly decreased

🞎 Strongly decreased

E4b. Would you say this change is related to your participation in the garden?

🞎 Yes

🞎 Yes, in part

🞎 No

E5a. Compared to when you started the garden (more than a year ago now), would you say that your life satisfaction has changed?

🞎 Strongly increased

🞎 Slightly increased

🞎 Did not changed

🞎 Slightly decreased

🞎 Strongly decreased

E5b. Would you say this change is related to your participation in the garden?

🞎 Yes

🞎 Yes, in part

🞎 No

E6a. Compared to when you started the garden (more than a year ago now), would you say that your satisfaction with your social relationships has changed?

🞎 Strongly increased

🞎 Slightly increased

🞎 Did not changed

🞎 Slightly decreased

🞎 Strongly decreased

E6b. Would you say this change is related to your participation in the garden?

🞎 Yes

🞎 Yes, in part

🞎 No

E7. Do you have any specific comments or clarifications to make?

................................................................................................................................................... ............................................................................................................................................................................................................................................................................................................................................................................................................................................................
