# Supplementary material for: Improving lifestyles sustainability through community gardening: results and lessons learnt from the JArDinS quasi-experimental study
Source: BMC Public Health. 2020 Nov 26;20:1798. doi: 10.1186/s12889-020-09836-6 (PMC7690132; doi:10.1186/s12889-020-09836-6)
Supplement: Supplementary file 3 — Additional file 3. Group differences and time effect of lifestyles components among active gardeners (≥ 1 visit per month throughout the year, n = 37) and paired non-gardeners. [file 12889_2020_9836_MOESM3_ESM.docx]

**Additional file 2.** Group differences and time effect of lifestyles components among active gardeners (≥ 1 visit per month throughout the year, n=37) and paired non-gardeners^a^

| **Sustainability components, means (SD)^b^** | **Model^c^** | **Gardeners (n = 37)** | | **Non-gardeners (n = 37)** | | **Group P-Value** | **Time P-Value** | **Group* Time P-Value** |
| --- | --- | --- | --- | --- | --- | --- | --- | --- |
|  |  | **t0** | **t1** | **t0** | **t1** |  |  |  |
| **Health dimension** |  |  |  |  |  |  |  |  |
| *Healthiness of household’s food supply*^d,e^ |  |  |  |  |  |  |  |  |
| Fruit & Vegetables^f^ (g/d/p) | B | 403.6 (226.5) | 405.3 (254.7) | 458.8 (312.3) | 477.3 (290.1) | 0.126 | 0.415 | 0.540 |
| MAR (% adequacy/2000kcal) | B | 75.8 (7.2) | 75.8 (8.5) | 75.3 (8.1) | 76.7 (6.1) | 0.811 | 0.479 | 0.518 |
| MER (% excess/2000kcal) | B | 97.3 (21.0) | 95.3 (26.8) | 104 (30.4) | 96.7 (30.3) | 0.819 | 0.204 | 0.469 |
| HPI [range: 0-15] | B | 8.6 (1.9) | 8.8 (1.9) | 9.2 (2.2) | 9.1 (1.7) | 0.208 | 0.908 | 0.644 |
| *Physical activity* |  |  |  |  |  |  |  |  |
| PAEE (kJ/kg/d) | A | 41.9 (10.9) | 40.2 (13.5) | 43.5 (14.2) | 40.3 (16.4) | 0.227 | 0.056 | 0.699 |
| Inactivity (h/d) | A | 569 (93.6) | 586.7 (85.3) | 557.1 (87.0) | 595.6 (84.5) | 0.605 | **0.001** | 0.286 |
| Low-intensity activity (h/d) | A | 165.4 (43.8) | 157.5 (48.3) | 160.9 (45.7) | 147.3 (44.6) | 0.597 | **0.043** | 0.605 |
| Moderate-to-vigorous intensity activity (h/d) | A | 106.4 (43.8) | 93.3 (41.2) | 113.8 (46.0) | 99.1 (54.4) | 0.312 | **0.002** | 0.985 |
| BMI (kg/m^2^) | D | 22.7 (2.9) | 23.1 (2.9) | 23.7 (4.1) | 23.8 (4.1) | 0.625 | 0.089 | 0.204 |
| WEMWBS [range: 14-70] | C | 51.6 (6.7) | 52.3 (6.6) | 52.6 (6.9) | 51.9 (5.6) | 0.250 | 0.926 | 0.248 |
| UCLA Loneliness Scale [range: 20-80] | C | 41.1 (10.8) | 39.6 (11.4) | 39 (8.8) | 39.4 (7.8) | 0.971 | 0.449 | 0.191 |
| **Environmental dimension** |  |  |  |  |  |  |  |  |
| High sensitivity to food waste, n (%) | C | 51.6 (4.2) | 51.4 (8.2) | 48.9 (7.6) | 50.9 (3.9) | 0.069 | 0.055 | 0.341 |
| Nature Relatedness Scale [range: 1-5] | C | 4.1 (0.4) | 4.1 (0.4) | 3.8 (0.5) | 3.9 (0.5) | **0.012** | 0.370 | 0.719 |
| *Environmental impact of household’s food supply*^d,e^ |  |  |  |  |  |  |  |  |
| GHGE (in g CO_2_eq/2000kcal)^f^ | B | 2943.9 (759.8) | 3162.3 (986.7) | 3304.8 (988.9) | 3270.1 (937.2) | 0.320 | 0.374 | 0.342 |
| Atmospheric acidification (in g SO_2_eq/2000kcal)^f^ | B | 31.2 (8.9) | 33.5 (11.6) | 38.7 (17.0) | 36.0 (11.4) | 0.238 | 0.796 | 0.201 |
| Marine eutrophication (in g Neq/2000kcal)^f^ | B | 11.8 (3.0) | 12.6 (3.4) | 13.6 (4.2) | 13.2 (3.2) | 0.081 | 0.824 | 0.463 |
| Animal to plant protein ratio of household food supply^f^ | B | 56.2 (14.9) | 55.6 (16.6) | 61.5 (16.6) | 60.5 (14.2) | 0.276 | 0.536 | 0.862 |
| **Economic dimension** |  |  |  |  |  |  |  |  |
| Household food expenditure (€/d/p)^d,e^ | B | 7.0 (3.0) | 7.0 (3.3) | 6.9 (3.1) | 7.4 (3.2) | 0.626 | 0.562 | 0.524 |
| *Expenditure share by food groups (%)*^d,e^ |  |  |  |  |  |  |  |  |
| Fruits & Vegetables | B | 26.1 (10.2) | 26.3 (10.7) | 27.8 (13.6) | 30.3 (15.7) | 0.180 | 0.146 | 0.208 |
| Starches | B | 10.4 (5.6) | 10.8 (5.6) | 8.6 (4.8) | 8.6 (4.2) | 0.108 | 0.701 | 0.661 |
| Meat, fish & Eggs | B | 18.5 (9.1) | 18.9 (9.7) | 21.4 (10.1) | 21.1 (11.2) | 0.325 | 0.886 | 0.838 |
| Dairy products | B | 11.2 (5.0) | 11.5 (4.7) | 11 (4.6) | 11.7 (5.1) | 0.956 | 0.371 | 0.745 |
| Mixed dishes^f^ | B | 9.2 (6.7) | 8.7 (6.7) | 7.4 (5.7) | 7.7 (7.9) | 0.112 | 0.405 | 0.902 |
| Sweet products | B | 10.4 (5.2) | 12.1 (8.5) | 11.2 (6) | 9.6 (5.5) | 0.443 | 0.977 | 0.062 |
| Added fats & seasonings^f^ | B | 4.5 (2.9) | 5.1 (2.6) | 3.2 (2.2) | 3.5 (2.5) | **0.007** | 0.237 | 0.383 |
| Beverages^f^ | B | 9.7 (6.2) | 7.6 (5.2) | 9.3 (7.5) | 7.5 (5.1) | 0.804 | **0.037** | 0.841 |

^a^ Abbreviations: GHGE: GreenHouse Gas Emissions; HPI: Healthy Purchase Index; MAR: Mean Adequacy ratio; MER: Mean Excess Ratio; PAEE: Physical activity energy expenditure; WEMBWS: The Warwick-Edinburgh Mental Wellbeing Scale; HPI: Healthy Purchase Index.

^b^ Unless specified.

^c^ Model A was adjusted on BMI and education level. Model B = Model A + percentage of meals consumed outside of the home. Model C = Model A + social desirability scale. Model D was adjusted on education level, percentage of meals consumed outside of the home and social desirability scale.

^d^ Variable measured at the household level and not at the individual one.

^e^ Including produce from the garden and foods from gifts or food aid. For food expenditure variables, a mean price was attributed to these foods (see method section).

^f^ Variable was log-transformed to improve normality.
