# Supplementary material for: Improving lifestyles sustainability through community gardening: results and lessons learnt from the JArDinS quasi-experimental study
Source: BMC Public Health. 2020 Nov 26;20:1798. doi: 10.1186/s12889-020-09836-6 (PMC7690132; doi:10.1186/s12889-020-09836-6)
Supplement: Supplementary file 4 — Additional file 4. Group differences and time effect of lifestyles components of gardeners who did not dropped out the garden during the year (n = 50) and paired non-gardeners. [file 12889_2020_9836_MOESM4_ESM.docx]

**Additional file 3.** Group differences and time effect of lifestyles components of gardeners who did not dropped out the garden during the year (n=50) and paired non-gardeners^a^

| **Sustainability components, means (SD)^b^** | **Model^c^** | **Gardeners (n = 50)** | | **Non-gardeners (n = 50)** | | **Group P-Value** | **Time P-Value** | **Group* Time P-Value** |
| --- | --- | --- | --- | --- | --- | --- | --- | --- |
|  |  | **t0** | **t1** | **t0** | **t1** |  |  |  |
| **Health dimension** |  |  |  |  |  |  |  |  |
| *Healthiness of household’s food supply*^d,e^ |  |  |  |  |  |  |  |  |
| Fruit & Vegetables^f^ (g/d/p) | B | 404.1 (240.5) | 415.7 (243.9) | 468.2 (294.1) | 510.6 (317.4) | **0.012** | 0.121 | 0.660 |
| MAR (% adequacy/2000kcal) | B | 76.5 (6.8) | 76.5 (7.0) | 76.4 (7.4) | 77.5 (6.4) | 0.369 | 0.500 | 0.514 |
| MER (% excess/2000kcal) | B | 97.4 (19.8) | 93.1 (20.1) | 98.7 (26.2) | 96.4 (28.5) | 0.854 | 0.212 | 0.708 |
| HPI [range: 0-15] | B | 8.5 (2.0) | 8.9 (1.9) | 9.2 (2.2) | 9.3 (1.8) | **0.028** | 0.213 | 0.388 |
| *Physical activity*^g^ |  |  |  |  |  |  |  |  |
| PAEE (kJ/kg/d) | A | 44.3 (13.7) | 41.4 (12.1) | 42.5 (12.9) | 39.7 (14.7) | 0.838 | **0.023** | 0.965 |
| Inactivity (h/d) | A | 558.2 (77.4) | 581 (80.3) | 558 (95.5) | 586.6 (83.6) | 0.898 | **< 0.001** | 0.468 |
| Low-intensity activity (h/d) | A | 164.1 (46.5) | 160.4 (50.9) | 164.2 (58.3) | 149.4 (45.7) | 0.889 | **0.040** | 0.224 |
| Moderate-to-vigorous intensity activity (h/d) | A | 113 (47.5) | 96.4 (38.6) | 108.7 (43.2) | 96.8 (49.7) | 0.682 | **< 0.001** | 0.688 |
| BMI (kg/m^2^) | D | 22.4 (3.2) | 22.7 (3.2) | 23.6 (4.0) | 23.7 (3.9) | 0.226 | **0.037** | 0.284 |
| WEMWBS [range: 14-70] | C | 51.8 (6.5) | 51.8 (6.1) | 51.7 (6.6) | 51.3 (5.4) | 0.056 | 0.754 | 0.698 |
| UCLA Loneliness Scale [range: 20-80] | C | 41.3 (10.1) | 40.6 (10.8) | 39.3 (9.8) | 39.9 (8.8) | 0.938 | 0.898 | 0.372 |
| **Environmental dimension** |  |  |  |  |  |  |  |  |
| High sensitivity to food waste, n (%) | C | 50.3 (5.2) | 50.2 (7.9) | 49.8 (7.3) | 51.4 (4.1) | 0.669 | 0.096 | 0.416 |
| Nature Relatedness Scale [range: 1-5] | C | 4.1 (0.4) | 4 (0.4) | 3.9 (0.5) | 3.9 (0.5) | 0.080 | 0.428 | 0.188 |
| *Environmental impact of household’s food supply*^d,e^ |  |  |  |  |  |  |  |  |
| GHGE (in g CO_2_eq/2000kcal)^f^ | B | 3054.6 (812.1) | 3136.6 (935.8) | 3295 (883.9) | 3277.9 (915.2) | 0.238 | 0.843 | 0.679 |
| Atmospheric acidification (in g SO_2_eq/2000kcal)^f^ | B | 33 (11.7) | 33.8 (11.3) | 37.5 (15.3) | 35.7 (12.5) | 0.379 | 0.817 | 0.339 |
| Marine eutrophication (in g Neq/2000kcal)^f^ | B | 12 (3.0) | 12.7 (3.9) | 13.6 (3.8) | 13.1 (3.0) | 0.083 | 0.737 | 0.168 |
| Animal to plant protein ratio of household food supply^f^ | B | 57.3 (14.7) | 56.9 (16.2) | 60.5 (15.3) | 58.8 (15.2) | 0.612 | 0.301 | 0.530 |
| **Economic dimension** |  |  |  |  |  |  |  |  |
| Household food expenditure (€/d/p)^d,e^ | B | 7.1 (3.0) | 6.7 (3.0) | 6.9 (3.1) | 7.4 (3.2) | 0.472 | 0.913 | 0.128 |
| *Expenditure share by food groups (%)*^d,e^ |  |  |  |  |  |  |  |  |
| Fruits & Vegetables | B | 25.7 (11.1) | 26.8 (10.2) | 28.7 (12.9) | 30.9 (15.4) | 0.050 | 0.100 | 0.583 |
| Starches | B | 10 (5.3) | 10.7 (5.2) | 9.3 (4.8) | 8.6 (3.8) | 0.155 | 0.963 | 0.071 |
| Meat, fish & Eggs | B | 18.6 (9.2) | 19.3 (10.0) | 20.0 (9.8) | 20.0 (10.4) | 0.690 | 0.750 | 0.816 |
| Dairy products | B | 11.8 (5.0) | 11.4 (4.5) | 11.0 (4.3) | 11.2 (5.3) | 0.428 | 0.758 | 0.562 |
| Mixed dishes^f^ | B | 9.8 (6.6) | 8.7 (6.4) | 8.3 (6.4) | 8.2 (7.8) | 0.061 | 0.182 | 0.866 |
| Sweet products | B | 10.4 (5.6) | 10.9 (6.3) | 10.7 (5.6) | 10.2 (5.9) | 0.897 | 0.997 | 0.460 |
| Added fats & seasonings^f^ | B | 4.3 (2.7) | 5.1 (2.6) | 3.2 (1.9) | 3.0 (2.0) | **< 0.001** | 0.488 | 0.057 |
| Beverages^f^ | B | 9.4 (5.9) | 8.2 (5.7) | 8.8 (6.8) | 7.8 (6.1) | 0.527 | 0.053 | 0.814 |

^a^ Abbreviations: GHGE: GreenHouse Gas Emissions; HPI: Healthy Purchase Index; MAR: Mean Adequacy ratio; MER: Mean Excess Ratio; PAEE: Physical activity energy expenditure; WEMBWS: The Warwick-Edinburgh Mental Wellbeing Scale; HPI: Healthy Purchase Index.

^b^ Unless specified.

^c^ Model A was adjusted on BMI and education level. Model B = Model A + percentage of meals consumed outside of the home. Model C = Model A + social desirability scale. Model D was adjusted on education level, percentage of meals consumed outside of the home and social desirability scale.

^d^ Variable measured at the household level and not at the individual one.

^e^ Including produce from the garden and foods from gifts or food aid. For food expenditure variables, a mean price was attributed to these foods (see method section).

^f^ Variable was log-transformed to improve normality.

^g^ Participants with less than 3 valid days (≥ 10 h of wearing the accelerometer wearing during daytime) were excluded from the analysis resulting in 50 gardeners and 49 controls at t0, and 48 gardeners and 48 controls at t1.
